# Experiences of patients with fibromyalgia at a Finnish Health Centre: A qualitative study

**DOI:** 10.1080/13814788.2022.2085683

**Published:** 2022-06-21

**Authors:** Aleksi Varinen, Tiina Vuorio, Elise Kosunen, Tuomas H. Koskela

**Affiliations:** a Department of General Practice, Faculty of Medicine and Health Technology, Tampere University, Tampere, Finland; b Finnish Student Health Service, Turku, Finland; c Centre for General Practice, Tampere University Hospital, Tampere, Finland

**Keywords:** Fibromyalgia, qualitative study, focus group, continuity of care, doctor-patient relationship

## Abstract

**Background:**

Fibromyalgia is a functional syndrome. Despite recent findings, there is still considerable uncertainty about its diagnostic process.

**Objectives:**

This study aimed to explore patients’ experiences with fibromyalgia during the diagnostic process in primary health care. Moreover, we tried to determine how diagnostic consultation could be improved.

**Methods:**

This study is based on data from patients with fibromyalgia in a primary health care study conducted in Nokia, Finland. Patients with fibromyalgia were identified from electronic medical records. Focus-group participants with fibromyalgia diagnoses were selected using a purposive sampling method to gather a maximum variation sample. Qualitative thematic analysis was used for the coded data from four focus-group discussions in 2018. A description of the coding tree was provided and researchers organised the codes. Finally, all researchers identified themes from the data.

**Results:**

The main unifying entities were the uncertainty and contradictions fibromyalgia patients faced on several occasions. Physicians sometimes offered other diagnoses – like depression – as an explanation for the symptoms, or used repetitive tests to eliminate other possible diagnoses. Furthermore, patients expressed their wishes for a holistic, empathetic, and up-to-date approach to their symptoms.

**Conclusion:**

In our interviews, a good doctor-patient relationship and continuity of care were necessary, as were the physician’s attitude and knowledge of fibromyalgia. Our findings also suggest avoiding repeated or unnecessary rule-out tests and the overdiagnosis of psychiatric disorders is necessary.

KEY MESSAGES

- Several patients had faced uncertainty and contradictions regarding fibromyalgia syndrome, and at least some of these feelings appeared to originate from physicians’ varying attitudes and knowledge.
- Patients valued an excellent doctor-patient relationship and continuity of care.
- There is a need to develop the diagnostic process and treatment of fibromyalgia in primary care.

## Introduction

In the eyes of many physicians, fibromyalgia is still a questionable disease entity, partially because of its mysterious pathophysiology [1]. It is a functional syndrome characterised by central sensitisation [2]. In addition to pain, patients can experience fatigue, poor sleep quality, cognitive problems, and various other symptoms [3].

There are several challenges relating to a fibromyalgia diagnosis. Various diagnostic criteria have been developed chiefly for research purposes but these criteria are also used for diagnostic purposes [4]. These criteria also received criticism over the confusion that the role of the tender points caused [5]. Based on the criticism, the ACR 2010 criteria were developed to give physicians an alternative that did not require tender point examination [5]. The original criteria also included the stipulation that the symptoms cannot be explained by any other disorder but this was later removed [6]. In clinical practice, laboratory tests are unnecessary for a fibromyalgia diagnosis but clinical guidelines recommend them to rule out diseases (such as anaemia or hypothyroidism) causing fibromyalgia-like symptoms [7].

Previous studies show there is significant under- and overdiagnosis concerning fibromyalgia, and the diagnosis often fails to provide an adequate clinical concept fit to the experience of the illness [8,9]. Indeed, many symptoms of fibromyalgia overlap with other diseases, which might lead to the overdiagnosis of subclinical manifestations of these diseases, as rule-out laboratory tests are often ordered during the diagnostic process [10,11]. It also appears likely that some physicians do not accept the diagnosis of fibromyalgia and are not willing to diagnose it even if the patient has typical symptoms, which may result in underdiagnosis [9]. In part, the reluctance might be caused by the belief that by not setting the diagnosis, they will avoid the overdiagnosis and medicalisation of fibromyalgia symptoms [12]. Furthermore, the diagnosis of fibromyalgia often fails to provide a valid explanation of the patient’s symptoms [8]. On the other hand, some physicians find the treatment of fibromyalgia frustrating because of the difficulty in controlling the symptoms and the patient’s emotional response, and there are no pharmacological interventions that are suitable for all patients and all symptoms [13,14].

A meta-ethnography study focussed on the diagnostic experiences of patients with fibromyalgia found that patients had often searched for a long time in their health care for the correct diagnosis [15]. A meta-synthesis of qualitative studies from illness experiences of fibromyalgia reported the same finding and this period before the diagnosis was difficult for patients [16]. In another study, patients found the overlapping symptoms confusing and this caused uncertainty and doubts regarding the accuracy of the diagnosis [17]. When the diagnosis was set, it validated and made sense of the symptoms. However, this relief was short and patients began to question the validity of the diagnosis and medical authority [15]. In addition, the invisibility of symptoms raised questions regarding the patients’ credibility [16].

To conclude, from previous studies, it is known that the time before a diagnosis is burdensome from the patient’s perspective. Diagnosis offers some help and validates the symptoms but usually, this does not last long, as the patient realises that, life has changed forever due to fibromyalgia. However, most patients eventually learn to cope with the symptoms [15,16,18,19]. These coping mechanisms vary from one patient to the next, and patients also desire longer consultation times in primary health care, continuity of care, and correct information on the aetiopathogenesis of fibromyalgia [16,20,21].

To the best of the authors’ knowledge, there are no previous qualitative studies on the diagnostic experiences of fibromyalgia patients in terms of the under- and overdiagnosis of fibromyalgia or the comorbidities of fibromyalgia from the patients’ viewpoint. This study aims to determine the patients’ experiences with fibromyalgia during the diagnostic process, especially in terms of possible diagnostic inaccuracies, and to ascertain how diagnostic consultation could be improved.

## Materials and methods

The research team and reflexivity: Aleksi Varinen (AV, male, GP) and Tiina Vuorio (TV, female, GP) conducted the focus-group interviews. In addition, Tuomas Koskela (TK, male, GP, professor of general practice) and Elise Kosunen (EK, female, professor of general practice) also designed the study and interpreted the data. AV works as a clinical lecturer at Tampere University, and at the time of the interviews, TV worked as a clinical lecturer at Turku University. AV had established prior contact with all the patients in one research appointment during the previous phase of the fibromyalgia study. Three of the patients were formerly patients of AV at Nokia Health Centre. However, they were not AV’s patients during or after the study, and their fibromyalgia diagnosis was set before they became AV’s patients. Therefore, the researchers were not involved in the diagnostic process of the study patients. All patients had previously received an information letter on this study and the researchers’ interest in the topic.

The methodological orientation of this study is a thematic analysis based on the phenomenological theory of the description of the participants' personal subjective experiences [22]. The participants were selected using purposive sampling. The study is based on data from patients with fibromyalgia participating in the Finnish Primary Health Care study conducted at Nokia Health Centre. Fibromyalgia patients were sourced from electronic medical records. The inclusion criteria were the ICD-10 code corresponding to fibromyalgia (M79.7) or a diagnosis of fibromyalgia in the patient records with some other code (M79.0, M25.5, R52.9, and M79.1). Altogether, 208 patients with fibromyalgia were identified. An information letter containing five questionnaires was sent to the patients. A GP’s (AV) appointment was scheduled for the 103 patients who responded to the letter. Altogether, 96 patients had fibromyalgia according to the ACR 2010 criteria and seven patients did not meet the ACR 2010 criteria for fibromyalgia at the time of the study. During the appointment, patients who met the criteria had the chance to ask questions about the syndrome.

Finally, for the qualitative part of the study, patients with fibromyalgia were selected using the purposive sampling method. AV selected the patients based on the information gathered from the previous stage of the fibromyalgia study to create a maximum variation sample. The criteria used for this were age, gender, educational level, and years since the original diagnosis of fibromyalgia. All patients were initially approached by mail to obtain written consent for the fibromyalgia study. Patients selected for the focus groups were contacted by telephone by AV. Initially, the four focus-group sessions were planned based on the estimate that four groups would be enough to achieve data saturation [23]. Each group was planned to contain five patients because of the topic’s complexity and possibly controversial content [23]. Four patients declined the invitation to participate in the focus-group interviews due to timetable constraints. At this point, 20 patients were selected for the study, however, two patients cancelled their appointment at the last minute.

As a result, the study sample consisted of 18 patients divided into four focus groups (two groups with five participants and two with four participants). The participants were referred to the different groups according to their age to keep the conversation pace comfortable for all participants [24]. The interviews took place in the Nokia Health Centre auditorium on 19–20 March 2018. No one else was present besides the participants and researchers. The characteristics of the participants are shown in Table 1. The age of the patients and the years since the diagnosis are shown in Supplementary Table 1.

**Table 1.** Demographic features.

Characteristic	Participants (*n* = 18)
Age (mean & standard deviation, SD)	54.7 ± 15.5
Gender (*n*)	
Female	15
Male	3
Educational level (*n*)	
Primary school	6
Upper secondary school or vocational school	11
University or polytechnic	1
Employment status (*n*)	
Engaged in working life (full- or part-time)	5
Unemployed	2
Unable to work (absence due to illness or disability pension)	9
Old-age pension	2
FM severity: PSD^a^ score (mean & SD)	22.5 ± 4.0
Years with FM^b^ (mean & SD)	13.8 ± 9.9
FM diagnosis set in (*n*)	
Primary health care	6
Secondary health care	12
Number of other diagnoses (mean & SD)	1.8 ± 1.2
Regular medication (*n*)	
Yes	9
No	9
Number of GP visits last year (mean & SD)	3.5 ± 2.0

^a^The PSD score is derived from the American College of Rheumatology (ACR) 2010 diagnostic criteria as the widespread pain index and symptom severity scale are combined into one index ranging from 0 to 31. A patient who fills the ACR2010 criteria for FM will always have at least 12 points from the PSD score. The PSD score is strongly related to somatic symptom severity [29].

^b^The time since the FM diagnosis ranged from 2 to 32 years.

The interview guide (Supplementary File) was available to the interviewers. A preliminary interview guide was developed based on previous research findings, and all of the researchers participated in formulating it to facilitate the data collection. The three main questions considered the patients’ desires for their treatment in primary health care, how the diagnostic process of fibromyalgia started, and the patients’ thoughts about the diagnostic tests used. The first session was also a pilot for the interview guide and no modifications were made based on that session.

The interviews were recorded (by audio). Field notes were also made during the sessions. The continuity of questioning was maintained by following the interview guide in every focus-group interview. Four sessions were carried out and after that, the researchers agreed that data saturation had been reached based on the field notes and no more focus-group interviews were needed. All participants participated equally, except for one patient who had suffered a stroke earlier; this slightly affected her ability to speak as fluently as the other patients. The duration of the interviews ranged from 61 to 95 min.

The data were coded according to the thematic analysis process [23]. We identified subordinate codes within group interviews and arranged them into more prominent themes across the interviews. Initially, 61 codes were generated. Due to overlaps, some codes were unified, resulting in 55 codes altogether (Supplementary Table 2). Finally, seven themes were formulated. Researchers used back-and-forth translation to verify that the citations were translated adequately. COREQ and SRQR checklists were used in reporting this study. The demographic features of the study population are presented in Table 1.

## Results

The following seven themes (Table 2) emerged from the data:

**Table 2.** Main themes from the focus-group interviews.

Searching for a reason for their illness
Prolonged diagnostic process
Contradictory and suspicious thoughts regarding the diagnosis
Need for compassion and understanding
The importance of the doctor-patient relationship
Illness and identity
Conceptions of the treatment

### Prolonged diagnostic process

The patients felt that physicians ordered diagnostic tests for rheumatoid arthritis and other various somatic diseases, but as they were negative, receiving the fibromyalgia diagnosis was a slow process:

*‘Laboratory tests for rheumatoid arthritis had been taken quite regularly since I was 14 or 15 years old, and nothing had ever been found’. (Woman, 40 years, 16 years since the diagnosis)*

In many cases, the participants mentioned that the rheumatologist had set the final diagnosis of fibromyalgia. In addition, the patients felt that on several occasions, physicians considered the symptoms of fibromyalgia (e.g. fatigue) to be a sign of depression, even though the patients did not feel their mood was low:

*‘When I went to clinical examinations for my pain, the only diagnosis that I got was depression’. (Woman, 33 years, 4 years since the diagnosis)*

Some participants felt that physicians did not set the diagnosis and tried to provide treatment advice instead:

*‘At some point I was getting frustrated and the doctor just said that there is nothing wrong with me: a person can have pain that comes from an unknown origin’. (Man, 62 years, 3 years since the diagnosis)*

Sometimes patients thought that physicians knew more than they revealed, but they were not allowed to tell everything, especially if there was a lack of sound scientific evidence:

*‘But I think that many doctors know more, but they cannot – is it their ethics or what – but they just can’t tell you what to do even if it would ease the symptoms. They have to use that medical jargon’. (Woman, 40 years, 16 years since the diagnosis)*

Uncertainty was more evident if the patients were told they had a fibromyalgia-like syndrome or if the patients were atypical (e.g. young or male). The role of tender points was also confusing, as many patients had more severe pain elsewhere. Some participants had different pain syndrome diagnoses (e.g. fibromyalgia and chronic pain syndrome), which was also considered confusing.

### Contradictory and suspicious thoughts regarding the diagnosis

This theme included negative attitudes toward the diagnosis of fibromyalgia as well as from the patient’s, physician’s, and society’s side because of the negative stigma:

*‘But at the moment when the diagnosis was set, the doctor said to me that you have to understand that this is a disease which is not taken seriously. So shall I set this diagnosis or not? And I replied that you have to do it if the symptoms match’. (Woman, 65 years, 9 years since the diagnosis)*

Patients often felt it hard to accept the diagnosis of fibromyalgia, and they would have wanted more tests to find out what was wrong with them. Sometimes the patients received only the diagnosis but no treatment or instructions on how to cope with the situation:

*‘It was actually the only thing that I did not want – the diagnosis – I wanted instructions for treatment’. (Woman, 49 years, 13 years since the diagnosis).*

On the one hand, some patients argued that fibromyalgia did not explain all of their symptoms. On the other hand, some patients reported that they were diagnosed quickly, as the GP seemed familiar with the syndrome and a few patients already suspected they had fibromyalgia.

In some cases, the participants felt that the physician had kept the diagnosis secret from them or made them decide whether they wanted the fibromyalgia diagnosis to be written in the medical record:

*‘I felt so embarrassed about that disease, because the occupational health doctor said to me, that do you want… are you really sure that you want me to put this diagnosis in your medical records’. (Woman, 65 years, 9 years since the diagnosis).*

Furthermore, many patients felt that they did not go to see their GP because of the fibromyalgia symptoms but due to other symptoms or because they did not know if the symptoms resulted from fibromyalgia. They thought it was tough to tell which condition was causing the symptoms, especially when they had many comorbidities:

*‘I have gout and other diseases. And osteoarthritis. I have several diseases that cause pain. Fibromyalgia is not the only one. You don’t always know which the pain is from’. (Woman, 50 years, 10 years since the diagnosis)*

### Searching for a reason for the illness

This theme included the patients’ thoughts about heritability and psychological factors (e.g. adverse life events) that could trigger fibromyalgia and thoughts about defects in their body (e.g. the hypermobility of joints) predisposing them to fibromyalgia:

*‘I am the third generation of women with this disease’. (Woman, 49 years, 13 years since the diagnosis)*

*‘I have guessed that I have it, because we have that in my family and the symptoms are the same’. (Man, 59 years, 3 years since the diagnosis)*

*‘I have also too straight a spine’. (Woman, 49 years, 13 years since the diagnosis)*

### Lack of compassion and understanding

A lack of empathy and understanding of the effects of fibromyalgia were the main features of this theme. The participants felt that close relatives and health care workers did not understand them. Furthermore, they felt that physicians questioned their credibility because they did not look sick:

*‘Even strangers tell you that you look so lively and happy that how can you not go to work’. (Woman, 49 years, 13 years since the diagnosis)*

In addition, the patients reported that physical examinations were often painful and the physicians did not seem to understand this:

*‘When doctors say that checking blood pressure can’t hurt, and it hurt so much that I almost fainted’. (Woman, 47 years, 14 years since the diagnosis)*

Moreover, patients felt that there was a lack of understanding also from society, as the diagnosis of fibromyalgia does not entitle one to a disability pension. On the other hand, one patient described how she regained her credibility when she was granted a disability pension:

*‘When I got my disability pension, it did not make me healthy but I felt that my dignity was restored’. (Woman, 69 years, 27 years since the diagnosis)*

Furthermore, some patients felt that fibromyalgia was not taken into consideration in the care planning for other medical conditions:

*‘After the fibromyalgia diagnosis, when I have visited doctors for other symptoms, no one has considered that they may be from fibromyalgia’. (Woman, 65 years, 9 years since the diagnosis)*

On the contrary, some felt that physicians thought that every symptom they had derived from the fibromyalgia syndrome:

*‘It takes time to understand the symptoms and how they present. It takes time to know yourself and your symptoms’. (Woman 65 years, 9 years since the diagnosis)*

### The importance of the doctor-patient relationship

The doctor-patient relationship was essential for the patients. Additionally, patients appreciated it if the physician was familiar with the treatment of fibromyalgia:

*‘If you have a good doctor, he understands the correct treatment for you’. (Woman, 40 years, 16 years since the diagnosis)*

Listening and understanding the condition was the key element for a good relationship. Still, many physicians seemed to lose interest after setting the diagnosis and did not give any instructions for self-treatment. Patients also reported that the physician they saw changed all the time and the consultations were too short:

*‘The consultation time is short. Or if you get on-call appointment, then it is even shorter’. (Woman, 33 years, 4 years since the diagnosis)*

Furthermore, the patients desired consultations where specialists and their GP would plan the treatment together:

*‘Sometimes you hope that there would be some joint consultation with your GP and the rheumatologist or some other specialist’. (Man, 62 years, 3 years since the diagnosis)*

Some patients also recognised the limitations of the effective treatment methods for fibromyalgia.

### Illness and identity

The patients stated that when they realised there was no curative treatment for fibromyalgia they were forced to adopt a new identity. GPs often tried to provide reassurance by pointing out that the condition is not malignant. This, however, did not always work:

*‘Doctors often try to comfort you that this does not kill you. But I think that it is not comforting when you have forty or fifty years left to live and you know that the pain is not going anywhere’. (Woman, 49 years, 13 years since the diagnosis)*

Some patients felt that the illness burden of fibromyalgia was very high and experienced desperation due to the fact there is no cure for the syndrome:

*‘I have noticed that some older patients would rather have cancer, which can either be cured or it kills you. With fibromyalgia you have constant pain and no-one can help you’. (Woman, 49 years, 13 years since the diagnosis)*

On the one hand, the younger patients, in particular, described difficulties accepting the restrictions on their functional ability. On the other hand, older patients tended to accept fibromyalgia better as a part of their burden of illness. Some patients mentioned that for a while, they had even forgotten they had fibromyalgia when they got some more severe diseases:

*‘I had breast cancer a few years ago, and I have not visited the doctor because of fibromyalgia for about 20 years’. (Woman, 72 years, 25 years since the diagnosis)*

After accepting the diagnosis, it was easier to find coping strategies. Most patients said that listening to one’s body was the best advice for coping with the symptoms. As a result, they felt their life had to be organised according to the disease:

*‘On a holiday trip I have to schedule my life. Today I go walking and tomorrow I will lie by the pool. Makes you laugh (ironically), but that’s how it is’. (Woman, 40 years, 16 years since the diagnosis)*

### Conceptions of the treatment

The patients had different opinions on the effectiveness of different medications, nutritional guidance, physiotherapy, psychological interventions, cold therapy, peer support, and acupuncture:

*‘I was in a rheumatology clinic, and when they examined my tender points I yelled like a dying swan, and there was no question about the diagnosis, and then they started trying different medications: oral corticosteroids, etc. and nothing helped’. (Woman, 69 years, 32 years since the diagnosis)*

In general, some patients have experienced short-term benefits from corticosteroids and long-term benefits from exercise:

*‘Exercise helps, even if it is only ten minutes of walking’. (Woman, 65 years, 9 years since the diagnosis)*

More information on fibromyalgia and meaningful pursuits in daily life were also seen as beneficial:

*‘Maybe the best thing to do is to exercise, but not too much and try to clear your mind of pain-related issues to with meaningful daily life pursuits’. (Man, 62 years, 3 years since the diagnosis)*

Furthermore, the patients recognised the importance of sleep:

*‘You are more sensitive to pain if you do not sleep well’. (Man, 62 years, 3 years since the diagnosis)*

## Discussion

### Main findings

An excellent doctor-patient relationship and continuity of care were meaningful from the patients’ perspective, as were the physician’s attitude and knowledge of fibromyalgia. Furthermore, it is necessary to avoid repeated tests to eliminate other possible diagnoses and the overdiagnosis of psychiatric disorders in the diagnostic process of fibromyalgia.

We expected the patients to talk more about diagnostic procedures and the uncertainty relating to positive or negative findings. However, during the conversations, only repeated laboratory tests for ruling out rheumatoid arthritis and some other somatic diseases were raised. Instead of mentioning diagnostic procedures, the patients expressed their need to discuss the uncertainty and contradictions regarding the diagnosis and treatment of fibromyalgia they had faced on several occasions.

### Interpretation of the study results in relation to existing literature

From previous studies, it is known that patients face some uncertainty relating to the diagnosis and treatment of fibromyalgia. In our study, one potential source of the overdiagnosis of subclinical manifestations of other diseases was the physicians’ repetitive use of exclusion tests for other diseases. This might occur because the former diagnostic criteria for fibromyalgia syndrome required that other conditions possibly causing similar symptoms be ruled out. However, in the revised criteria, a diagnosis of fibromyalgia is valid irrespective of other diagnoses, though it is still vital to diagnose comorbidities that may cause similar symptoms [6]. Thus, fibromyalgia should not be seen as a rule-out diagnosis. On the other hand, sometimes, patients felt that physicians did not even set a preliminary diagnosis and provided treatment instructions instead.

The controversy is also present from the physicians’ point of view, as there are no specific diagnostic criteria for fibromyalgia developed especially for clinical work [5]. This and the difficulty of treating the symptoms can also confound physicians [14]. In our study, we discovered that patients experience this contradiction on many levels in health care, as well as in personal relationships and, more broadly, in society. Even though fibromyalgia is a medical diagnosis, the patients felt that some physicians did not believe that the patients benefitted from the diagnosis and were reluctant to set it. Epidemiological data from a previous study have shown similar results [9]. This may highlight that some physicians think the diagnosis is not helpful and by not setting the fibromyalgia diagnosis, they can avoid medicalisation [12]. Moreover, most of the patients had been diagnosed over a decade ago, which may reflect former attitudes towards fibromyalgia syndrome as well as problems with the former fibromyalgia criteria relating to clinical work [5]. On the other hand, our sample also included several patients who had been diagnosed only a few years ago, and these patients had similar experiences. Furthermore, various other diagnoses, such as depression, were offered as an alternative explanations for the symptoms. These alternative explanations might lead to the misdiagnosis or overdiagnosis of psychiatric conditions if the diagnostic guidelines for these diseases are not followed since there is some overlap in functional syndrome symptoms (e.g. poor sleep and cognitive problems) and depression.

Patients had also received different chronic pain diagnoses from other medical specialities (e.g. psychiatry), and the role of these alternative diagnoses was confusing to patients. A new unifying diagnostic construct of bodily distress syndrome including four symptom clusters (cardiopulmonary, gastrointestinal, musculoskeletal and general symptoms, or fatigue) has also been suggested instead of several speciality-specific functional syndrome diagnoses such as fibromyalgia, and this might reduce the diagnostic incoherence from that perspective [25].

From the physician’s viewpoint, it is also difficult to distinguish which symptoms derive from fibromyalgia and which might be symptoms of some other undiagnosed disease requiring further investigation. The continuity of care and an excellent doctor-patient relationship that patients emphasised during the interviews might help with this.

The patients’ treatment conceptions mainly were in line with the previous literature. Treatment should be individual and some medications may benefit some patients but cause severe side effects in others [13]. Several participants in our study found exercise beneficial, which is in line with findings from previous studies [2]. Previous literature and our findings also highlight the importance of the continuity of care and longer appointments in primary health care [20,21].

### Strengths and limitations

The strengths of this study include a comprehensive group of patients, including three male participants. The participants were of various ages and their symptoms vary from mild to severe. None of the participants had a current patient-doctor relationship with either of the interviewers, and the interviewers were not employees of the health centre at the time of the study. The questions presented at the interview were open-ended, and the participants started conversations without further encouragement. The interviews were carried out face-to-face. During the interviews, patients also brought up several themes identified in previous studies.

The inclusion of only one health centre is a limitation of this study. However, many patients had also had consultations in specialised and occupational health care and the patients had faced the same kind of attitudes and uncertainty in these contexts as at the health centre. Additionally, in the focus groups, not all the patients participated equally, and some might have left something unsaid.

### Implications for further studies and clinical practice

Based on our findings and other studies, there is a need to improve the diagnostic process for fibromyalgia in primary care to avoid the overdiagnosis of subclinical manifestations of diseases when rule-out tests are repeatedly used in the diagnostic process of fibromyalgia [9,10]. There also may be a need to adopt a concept for functional syndromes or bodily distress syndrome that is meaningful for both patients and GPs in order to clarify the role of overlapping functional syndrome or pain syndrome diagnoses and the symptoms they are causing. A holistic view and good communication skills are important factors in patient communication [8].

## Conclusion

Many patients have faced contradictions and uncertainty regarding fibromyalgia syndrome, and some of these feelings appear to originate from physicians’ varying attitudes and knowledge. On the other hand, a good doctor-patient relationship and continuity of care were highly valued: these core values of general practice need to be supported. Our findings suggest that it is necessary to develop the diagnostic process and treatment of fibromyalgia in primary care to avoid repeated or unnecessary rule-out tests and the overdiagnosis of psychiatric disorders based only on pain or functional syndrome symptoms.

Geolocation information: This study was conducted in the Pirkanmaa region of Finland.

## Supplementary Material

Supplementary Table 2

Supplementary Table 1

Supplementary Material: Interview Guide
