# Supplementary material for: Experiences of patients with fibromyalgia at a Finnish Health Centre: A qualitative study
Source: Eur J Gen Pract. 2022 Jun 21;28(1):157–64. doi: 10.1080/13814788.2022.2085683 (PMC9225685; doi:10.1080/13814788.2022.2085683)
Supplement: Supplementary Table 2 [file IGEN_A_2085683_SM5784.docx]

| Table 2. Main themes and codes from the focus group interviews. | | | | |
| --- | --- | --- | --- | --- |
| **Searching for a reason to their illness** | | |  |  |
| Thoughts about heritability | |  |  |  |
| Thoughts about defects or predisposing factors in their body | | | | |
| Thoughts about psychologic predisposing factors | | | |  |
| **Prolonged diagnostic process/journey** | | | | |
| Repeated diagnostic tests for rheumatoid arthritis or other diseases | | | | |
| Perception that rheumatologist sets the final diagnosis of FM | | | | |
| Depression as an alternative explanation for symptoms | | | | |
| The slowness of getting the diagnosis | | |  |  |
| Complicated diagnostic process due to the multiplicity of symptoms | | | | |
| Only treatment instructions instead of diagnosis | | | |  |
| Perception that physicians are not allowed to tell everything they know | | | | |
| Comprehensive and diverse symptoms | | |  |  |
| Perseverance needed to get the diagnosis and treatment | | | | |
| Patient’s suspicion of the FM diagnosis from the beginning | | | |  |
| **Contradictory and suspicious thoughts from the diagnosis** | | | | |
| Uncertainty in which symptoms are from FM | | | | |
| Uncertainty in which symptoms are from FM | | |  |  |
| Patient’s wishes for more diagnostic tests | | | |  |
| Patient’s suspicion of the FM diagnosis from the beginning | | |  |  |
| Quick recognition of FM | | | | |
| Only treatment instructions instead of diagnosis | |  |  |  |
| Perceived benefits from the diagnosis | | | |  |
| Negative attitude towards FM: the patient | | |  |  |
| Negative attitude towards FM: physicians | | |  |  |
| Negative attitude towards FM: the society | | |  |  |
| The difficulty of accepting the diagnosis | | |  |  |
| **Need for compassion and understanding** | | |  |  |
| Neglecting FM when planning treatment of other diseases | | |  |  |
| The lack of compassion and understanding from health care professionals | | | | |
| Pain caused by physical examination | | | | |
| Blaming the patient from the ineffectiveness of the treatment | | |  |  |
| **The importance of doctor-patient relationship** | | | | |
| The importance of physician’s knowledge off the treatment of fibromyalgia | | | |  |
| The emphasis of doctor-patient relationship | | | | |
| **Illness and identity** | | | |  |
| Despair caused by the fact that there is no cure for FM |  |  |  |  |
| Organizing life according to the disease | | | | |
| Perseverance needed to get the diagnosis and treatment | | |  |  |
| Impression of limited effect of different treatment options | | | | |
| Accepting the diagnosis and the illness | | | | |
| **Conceptions of the treatment** | | |  |  |
| Impression of limited effect of different treatment options | |  |  |  |
| The effectiveness or ineffectiveness of medications and unwillingness to use them | | | | |
| Perceptions concerning analgesics | | | | |
| Perceived positive effects of corticosteroids | | |  |  |
| Other treatment options besides analgesics | | | |  |
| Benefits and harms from nutritional guidance | | | |  |
| Benefits and harms from physiotherapy | | | |  |
| Benefits from psychological interventions | | |  |  |
| Significance of sleep | | |  |  |
| Reliance to alternative treatment |  |  |  |  |
| Benefits and negative effects from exposing to cold | | |  |  |
| Benefits of physical exercise | | | |  |
| Benefits of meaningful daily tasks | |  |  |  |
| Information about fibromyalgia | | |  |  |
| Benefits and negative effects from peer support | |  |  |  |
| Benefits and harms from acupuncture | | | |  |
| Wish of co-operation between primary and secondary health care | | |  |  |
|  | | | | |
