# Supplementary material for: Experiences of patients with fibromyalgia at a Finnish Health Centre: A qualitative study
Source: Eur J Gen Pract. 2022 Jun 21;28(1):157–64. doi: 10.1080/13814788.2022.2085683 (PMC9225685; doi:10.1080/13814788.2022.2085683)
Supplement: Supplementary Table 1 [file IGEN_A_2085683_SM5783.docx]

| Group 1 | Age | Years with FM diagnosis |
| --- | --- | --- |
|  | 47 | 14 |
|  | 49 | 13 |
|  | 40 | 16 |
|  | 50 | 10 |
| Group 2 |  |  |
|  | 59 | 3 |
|  | 61 | 19 |
|  | 62 | 3 |
|  | 66 | 9 |
|  | 65 | 9 |
| Group 3 |  |  |
|  | 32 | 2 |
|  | 34 | 13 |
|  | 33 | 4 |
|  | 30 | 2 |
| Group 4 |  |  |
|  | 72 | 32 |
|  | 72 | 25 |
|  | 74 | 15 |
|  | 69 | 32 |
|  | 69 | 27 |
