# Supplementary material for: Experiences of patients with fibromyalgia at a Finnish Health Centre: A qualitative study
Source: Eur J Gen Pract. 2022 Jun 21;28(1):157–64. doi: 10.1080/13814788.2022.2085683 (PMC9225685; doi:10.1080/13814788.2022.2085683)
Supplement: Supplementary Material: Interview Guide [file IGEN_A_2085683_SM5782.docx]

**Interview guide**

*Beginning of session*

(The interviewer introduces himself/herself and another interviewer): ‘Today we are going to discuss fibromyalgia. We aim to find out how you wish fibromyalgia to be treated in the future and what kind of thoughts you have on the diagnostic process of the syndrome. Do you have any question about the interview?’

The rules of the interview are reviewed. Cellphones are turned off. To monitor who is saying what, participants are asked to speak only one at a time. Participants are asked to raise their hand to indicate they want to speak. Participants are explained, that if the interviewer interrupts someone, it is because everyone deserves a chance to participate and the conversation stays on the subject.

Participants are informed that everything that is discussed is going to be confidential. After the conversation, the interviewers and two other members of the research group are going to summarise the discussion so that individual participants can not be recognised and compare themes that are come up to themes that have come up in the other focus groups.

‘At first, I will ask everybody to tell their name and age’ (participants are picked up in random order so they do not think they should say something based on their sitting order).

**Main theme 1: How do you wish fibromyalgia would be treated in the future (in primary health care)?**

**-**What kind of health care services do you wish there would be for fibromyalgia patients?

**-**What treatment or advice that you have received from health care has been most helpful?

**-**What has been the most useful thing to help you cope with fibromyalgia?

**Main theme 2: What do you think about internet-based therapy for fibromyalgia?**

-In which situation do you think it might be helpful?

-How should it be put into practice?

**Main theme 3: (Overdiagnosis)**

**What happened after your first visit to see a physician because of fibromyalgia symptoms?**

-What do you think about it now?

-Did symptoms worry you before the appointment?

**What do you think now about the diagnosis of fibromyalgia and diagnostic process?**

-Was something in the treatment beneficial?

-Did you think that something was unhelpful?

-Would you have liked that something in the diagnostic process would have been left out? (Can you give an example?)

-Did treatment reduce the burden that fibromyalgia caused you?

-Did you receive contradictory information about the cause of fibromyalgia? How did it feel like?

-Did you get different/contradictory diagnosis or treatment plans based on your symptoms?

-Were previous diagnoses overruled in the diagnostic process? How did it feel like?
